# Allele-specific expression at the androgen receptor alpha gene in a hybrid unisexual fish, the Amazon molly (*Poecilia formosa*)

**DOI:** 10.1371/journal.pone.0186411

**Published:** 2017-10-12

**Authors:** Fangjun Zhu, Ingo Schlupp, Ralph Tiedemann

**Affiliations:** 1 University of Evolutionary Biology/Systematic Zoology, Institute of Biochemistry and Biology, University of Potsdam, Potsdam, Germany; 2 Department of Biology, University of Oklahoma, Norman, Oklahoma, United States of America; Montana State University Bozeman, UNITED STATES

## Abstract

The all-female Amazon molly (*Poecilia formosa*) is the result of a hybridization of the Atlantic molly (*P*. *mexicana*) and the sailfin molly (*P*. *latipinna*) approximately 120,000 years ago. As a gynogenetic species, *P*. *formosa* needs to copulate with heterospecific males including males from one of its bisexual ancestral species. However, the sperm only triggers embryogenesis of the diploid eggs. The genetic information of the sperm donor typically will not contribute to the next generation of *P*. *formosa*. Hence, *P*. *formosa* possesses generally one allele from each of its ancestral species at any genetic locus. This raises the question whether both ancestral alleles are equally expressed in *P*. *formosa*. Allele-specific expression (ASE) has been previously assessed in various organisms, e.g., human and fish, and ASE was found to be important in the context of phenotypic variability and disease. In this study, we utilized Real-Time PCR techniques to estimate ASE of the *androgen receptor alpha* (*arα*) gene in several distinct tissues of Amazon mollies. We found an allelic bias favoring the maternal ancestor (*P*. *mexicana*) allele in ovarian tissue. This allelic bias was not observed in the gill or the brain tissue. Sequencing of the promoter regions of both alleles revealed an association between an Indel in a known CpG island and differential expression. Future studies may reveal whether our observed cis-regulatory divergence is caused by an ovary-specific trans-regulatory element, preferentially activating the allele of the maternal ancestor.

## Introduction

The Amazon molly (*Poecilia formosa*) is an all-female species with unusual unisexual reproduction, called gynogenesis. *P*. *formosa* originated from two bisexual ancestral species, the Atlantic molly, *P*. *mexicana*, and sailfin molly, *P*. *latipinna*, probably via a single hybridization event at least 120,000 years ago [1–9]. In this gynogenetic species, embryogenesis of the unfertilized diploid eggs must be triggered by heterospecific sperm from a male of a “donor” species [1]. Among others, males of the ancestral species, *P*. *mexicana* and *P*. *latipinna* provide sperm, although the genetic information of the sperm DNA will typically not be incorporated into the eggs of *P*. *formosa* [1,10,11]. As a hybrid species of *P*. *mexicana* and *P*. *latipinna*, *P*. *formosa* carries two sets of genomes from its ancestors. This fixed heterozygous state (sometimes called a frozen hybrid) of *P*. *formosa* was reported in several other studies [7,12,13] and raises important questions regarding gene expression and regulation.

The members of the nuclear receptor super-family, Androgen receptors (Ars), play a vital role in multiple biological functions by mediating androgens, including 11-ketotestosterone (11-KT) and testosterone (T). A teleost-specific whole-genome duplication (FSGD) [14] gave rise to two isoforms of Ar, Androgen receptor alpha (Arα) and Androgen receptor beta (Arβ). Most teleost fish analysed so far possess both paralogs of the Ar gene, but a few show only one gene copy which is orthologous either to Arα (e.g., *Haplochromis burtoni*) or to Arβ (e.g., *Danio rerio*) [15]. The coding region and expression of these two isoforms in *P*. *formosa* and its ancestors *P*. *mexicana* and *P*. *latipinna* were investigated in a previous study [15]. This study revealed heterozygous sites in the *androgen receptor alpha* (*arα*) coding region. The total mRNA expression of *P*. *formosa arα* did not show any statistically significant difference when compared to its ancestral species [15]. However, it was hitherto not evaluated how gene expression is regulated in a single cell with two genomes. Specifically, the gene expression was summed over the two alleles, but potentially differential expression between the two alleles was not investigated so far.

In a broader context, *P*. *formosa* provides a rare opportunity to study allele-specific expression (ASE) [16] and *cis*-regulatory evolution in a unisexual vertebrate species with clonally inherited hybrid genomes. In humans, e.g., up to 60% of the genes show significant ASE differences or allelic bias within and between individuals, populations [17–21], and tissues [22]. About 18% of all examined genes showed uneven expression between alleles in the liver of the triploid Japanese rice fish *Oryzias latipes* in a large-scale analysis using RNA-seq [23]. This ASE was regulated by either genetic or epigenetic putative allele-specific *cis*-regulatory factors [24,25]. CpG islands are larger than 200 bp DNA regions with high frequency of CpG sites and often occur near to promoter regions of genes [26]. In CpG islands, the percentage of guanine/cytosine is larger than 50% and the ratio of observed to expected CpG should be greater than 60% [27]. Previous studies have described extensive allele-specific effects of DNA methylation on gene expression [24,28]. Thus, mapping the methylation pattern of both CpG islands and other CpG sites can uncover the genetic basis of gene expression differences [29]. Several methods have been established to estimate allele-specific expression in various organisms based on Sanger sequencing, quantitative Real-Time PCR, or next generation sequencing (NGS) [30–36]. For ASE quantification at a single gene, Real-Time PCR has multiple advantages, with regard to sensitivity, specificity, and efficiency. Different strategies using Real-Time PCR with different primers, e.g., mismatch amplification mutation assay (MAMA), SYBR green with allele-specific primers, and fluorescence-labeled probes with primers, were employed in ASE and DNA SNPs allele frequency investigations [37–40]. An approach combining dual fluorescence probes labeled with minor groove binder (MGB) using TaqMan Real-Time PCR was reported in recent studies [41, 42]. Chen et al. developed such a quantification method to estimate the frequency of a resistance allele in pooled cotton aphid DNA samples, using the transformed fluorescence ratio k’ based on the raw fluorescence data with a predefined standard curve (for details see [42]). So far, this method has not been used on cDNA for estimating ASE.

We utilized the heterozygous site in *arα* CDS at position 1974 uncovered in a previous study [15] as the heterozygous marker to quantify ASE from several *P*. *formosa* tissues (ovary, brain, and gill) using a a modified version of the allele-specific qPCR assay described by Chen et al. [42]. Furthermore, we sequenced 670bp upstream of the ATG transcription start codon, aiming at encompassing the promoter region of *arα*, in order to look for allele-specific genetic variation in regulatory elements potentially related to ASE.

## Materials and methods

### Sampling, DNA, and RNA isolation

The founder fishes of the analysed *P*. *formosa* laboratory strain were collected from the Rio Purification (Barretal, Tamaulipas, Mexico) in 1993. Six laboratory-born *P*. *formosa* specimens were chosen for our study according to their similar weight, body size, and sexual development stage. All specimens were sacrificed on ice and all tissues were quickly excised and immediately frozen in liquid nitrogen and then stored in -80°C. The whole sampling process was limited to 15 minutes in order to prevent any impact on gene expression and it followed the international recognized guidelines and applicable national law (Tierschutzgesetz). The procedure was approved by the deputy of animal welfare in University of Potsdam. A combination of the Trizol (LifeTechnologies) and RNasey mini kit (Qiagen) method was implemented for total RNA isolation from ovary, brain, and gill tissues respectively [15]. 1ml Trizol was added into 10mg tissue immediately after it was taken out of the -80°C freezer. After homogenization with a Mini-Beadbeater (Glen Mills Inc.), 200μl chloroform was added into each sample. After centrifugation of the Trizol and chloroform mixture, the upper layer of the aqueous phase was transferred to the RNeasy mini kit column (Qiagen). Total RNA was isolated using a column and gDNA contamination was eliminated using RNase-Free DNase (Qiagen), following the instructions of the manufacturer. The tail fins of all six specimens were utilized for DNA extraction using a NucleoSpin^®^ Tissue kit (Macherey-Nagel). All steps followed the manufacturer’s protocol. The concentrations of DNA and RNA were determined using a NanoDrop 1000 Spectrophotometer (ThermoFisher Scientific). All six samples yielded sufficient DNA for gDNA analysis, and four samples yielded RNA of sufficient quality and quantity across all target tissues. RNA samples and DNA samples were stored at -80°C and -20°C, respectively, after isolation.

### Primer and probe design

**Fig 1:**
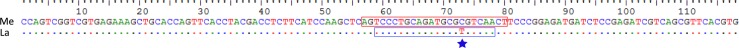
The probes and the investigated heterozygous site. The red rectangle indicates the VIC-labeled MGB probe for the *P*. *mexicana*-derived allele. The blue rectangle indicates the FAM-labeled MGB probe for the *P*. *latipinna*-derived allele. The blue star represents the heterozygous marker site. Abbreviation in figure, Me: *P*. *mexicana*-derived allele, La: *P*. *latipinna*-derived allele.

The heterozygous positions of *arα*, which can be used for ASE investigation and allele frequency estimation in gDNA, were detected in a previous study (GenBank: KP172521) [15]. One polymorphic position is located in exon 9 at site 1947 from the transcription start site (TSS) close to the exon-intron boundary, allowing us to design a primer crossing an exon-intron boundary to prevent the possibility of undesired gDNA amplification during ASE quantification. A TaqMan SNP assay with primers and probes was designed using Primer Express^®^ Software v3.0 (ThermoFisher Scientific). A 118 bp cDNA fragment was amplified with a forward primer (5’-CCAGTCGGTCGTGAGAAAGC-3’) and reverse primer (5’-CACGTGAACGCTGACGATCT-3’) at 60°C annealing temperature. Dual-labeled probes, 5’-VIC-AGTCCCTGCAGATGCGTGTCAACT-3’-MGB (Life Technologies) for the *P*. *mexicana*-derived allele and 5’-FAM-TCCCTGCAGATGCGCGTCAA-3’-MGB (Life Technologies) for the *P*. *latipinna*-derived allele were designed for ASE investigation (Fig 1).

As these primers cross an intron-exon, they were not suitable for the gDNA assessment. Therefore, a shorter DNA fragment (86 bp) was amplified for gDNA analysis with a forward primer (5’-CCTACGACCTCTTCATCCAAGCT-3’) and reverse primer (5’-CACGTGAACGCTGACGATCT-3’) at 60°C annealing temperature, applying the same dual-labeled probes that were used in the ASE investigation. The primers (*arα* Forward: AATTCATTGTGATCACGGAC; *arα* Reverse: CTTGGAGTTAAAGCAGAGTG; annealing temperature 54°C) for amplification of 670bp upstream the TSS (aimed at encompassing the putative promoter region) were designed with the Primer3 online tool (http://primer3.ut.ee), based on our previous *arα* coding region sequence (CDS) in *P*. *formosa* (GenBank: KP172521 and KP172522) and the genome sequence of *P*. *formosa* in the NCBI database (Accession: PRJNA237006).

### Reverse transcription, cloning, and sequencing

200ng total RNA (gDNA contamination removed using DNase) was reverse transcribed using the RevertAid First Strand cDNA Synthesis Kit (ThemoScientific) following the manufacturer’s protocol. Absence of undesired gDNA detection was verified via a negative control reaction (-RT) in which the reverse transcription enzyme was replaced by water. For the control, no amplification was detected in our highly sensitive Real-time PCR. The putative promoter region fragment of *arα* was amplified with MyTaq^TM^ DNA polymerase (Bioline) using DNA templates of corresponding specimens. Amplification parameters were as follows: 30μl volume according to manufacturer specifications, 94°C for 120s, 38 cycles 94°C for 20s, 60°C for 20s, and 72°C for 30s in cycler (Biometra). The fragments were checked for correct length by electrophoresis and purified with the NucleoSpin Gel and PCR Clean-up kit (Macherey-Nagel). Cleaned fragments were ligated with pCR™4-TOPO^®^ TA Vector and transformed into One Shot TOP10 Electrocomp™ *E*. *coli* (Life Technologies). After positive kanamycin selection, fragments were re-amplified using T3/T7 PCR with MyTaq^TM^ DNA polymerase (Bioline). The PCR products were purified by using Exonuclease I and Antarctic Phosphatase (New England BioLabs) and then sequenced on an ABI 3130xl automated sequencer (Applied Biosystems), using the BigDye Terminator v3.1 Cycle Sequencing Kit (Applied Biosystems). To facilitate the detection of the heterozygous state in any single *P*. *formosa* individual, at least 12 colonies for each *P*. *formosa* specimen were sequenced. Six colonies of each bisexual ancestral species, *P*. *mexicana* and *P*. *latipinna* were sequenced, respectively. The softwares Sequencher 5.2 (Gene Codes Corporation) and BioEdit [43] were employed for sequence alignment and visualization. The prediction of CpG islands was conducted using putative promoter regions sequences of *arα* with the online tool MethPrimer (http://www.urogene.org/cgi-bin/methprimer/methprimer.cgi) [29].

### Predefined plasmid mixture and standard curve

A 118 bp cDNA fragment containing the heterozygous site (site 1947, [15]) was amplified with the same primers as used for the ASE investigation. After gel purification, the cleaned fragments were cloned as previously mentioned. The plasmids (which contained either the *P*. *mexicana*-derived allele or the *P*. *latipinna*-derived allele) were extracted from the colony by heating at 95°C and purified by centrifuging at 12,000 x g for 5 minutes. The precise concentration of plasmid was measured using the Qubit® dsDNA HS Assay Kit (ThermoFisher Scientific). To create a reliable standard curve for precise ASE quantification, a series of standards with predefined allele mixtures was constructed by mixing plasmids carrying the respective alleles. In these mixtures, relative frequency x of the plasmid carrying the *P*. *mexicana*-derived allele was equal to 1.0, 0.95, 0.9, 0.8, 0.7, 0.6, 0.5, 0.4, 0.3, 0.2, 0.1, 0.05 and 0.0, respectively. The relative frequency of the *P*. *latipinna*-derived allele was 1-x.

### Allele-specific expression assay

A quantitative Real-Time TaqMan assay was performed in a 25μl reaction using an ABI 7500 Fast Real-Time PCR system (LifeTechnologies). Each reaction contained 800 nM forward and reverse primers, 200 nM MGB/fluorescence labeled probes, and the TaqMan Universal PCR Master Mix (LifeTechnologies). The Real-Time PCR reaction was carried out as follows: 95°C for 10min, 46 cycles 95°C for 15s, 60°C for 1min. Four *P*. *formosa* individuals were analyzed with this assay. Each of these biological samples was analyzed in triplicate. We quantified the relative proportions of the two alleles also in the gDNA of six *P*. *formosa* specimens with the same predefined standards and all the same reaction contents except for the forward primer which was different (see above). Different primers were necessary as the primers for ASE quantification used for cDNA crossed an exon-intron boundary, indeed to prevent the possibility of undesired gDNA amplification during ASE quantification. All the ASE reactions were jointly analyzed on a single plate, and also all allele frequency assessments in gDNA were performed on a single plate.

### The transformed fluorescence ratio K’

In our ASE study, we employed the allele frequency quantification method developed by Chen et al. [42]). Both raw fluorescence signals (probe-labeled fluorescence FAM and VIC for specific detection of the *P*. *latipinna*-derived and *P*. *mexicana* derived allele, respectively) were fitted into a four parametric sigmoid model (see equations below, [42]). To minimize parameter estimation bias caused by the variable number of cycles in the PCR plateau phase, a two-step sigmoid curve fitting method was utilized in our study (details see [42]). The model fitting was performed in R with the Mosaic package using the fitModel function [44].

When *b*_*fam*_ < *b*_*vic*_ (i.e., the FAM signal reached its maximum speed first),

$$R^{\prime }=0.5\times \frac{a_{fam}}{a_{vic}}\times (1+exp(\frac{-(b_{fam}-b_{vic})}{c_{vic}}))$$ (1)

Where:

*a*_*fam*_ represents the maximal height of the curve for fluorescence FAM (*P*. *latipinna*-derived allele).
*b*_*fam*_ represents the inflexion point of curve of *P*. *latipinna*-derived allele.
*c*_*fam*_ represents the slope of the curve of *P*. *latipinna*-derived allele.
*a*_*vic*_ represents the maximal height of the curve for fluorescence VIC (*P*. *mexicana*-derived allele).
*b*_*vic*_ represents the inflexion point of curve of *P*. *mexicana*-derived allele.
*c*_*vic*_ represents the slope of the curve of *P*. *mexicana*-derived allele.

When *b*_*fam*_ > *b*_*vic*_ (i.e., the VIC signal reaction reached its maximum speed first),

$$R^{\prime }=2\times \frac{a_{fam}}{a_{vic}}\times \frac{1}{1+exp(\frac{-(b_{vic}-b_{fam})}{c_{fam}})}$$ (2)

The transformed fluorescence ratio *k’* refers to the ratio of fluorescence *R’*

$$k^{\prime }=\frac{R\prime }{R^{\prime }+1}$$ (3)

## Results

### Genetic variation in the androgen receptor alpha putative promoter region

The 5’ end of the flanking regions of the transcription start site sequence of all three species were cloned and sequenced in our study (GenBank Accession Numbers: KX165280-KX165283; S1 Fig). Within this sequence, we identified a TATA box typical for promoters. There was one polymorphism and one indel out of 670 bp at site 77 and site 231, respectively, in the sequenced region distinguishing between the two bisexual ancestral species, each of which was fixed for a single allele. Consistent with its hybrid status, *P*. *formosa* exhibited both alleles. The prediction of CpG islands and CpG sites in the *arα* promoter is illustrated in S1 Fig. Interestingly, the indel site at 231 is situated in one CpG island region of *arα*.

### Transformed fluorescence ratio K’ and standard curve

**Fig 2:**
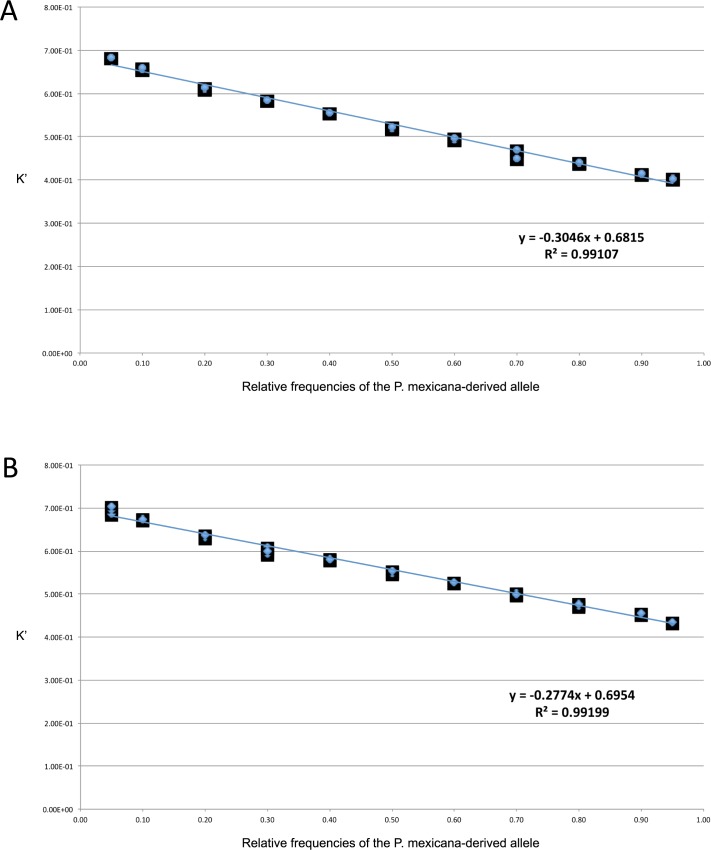
Standard curves of premixed plasmid preparations. The predefined relative frequencies x of the *P*. *mexicana-*derived allele (relative frequency of *P*. *latipinna*-derived allele amounts 1-x) were plotted against transformed fluorescence ratio K’ on cDNA level (A) and gDNA assessment (B). 0.5 on the x axis indicates an equal (i.e., 1:1) detection of both alleles.

To create a reliable standard curve for ASE and heterozygous allele frequency calculation, the transformed fluorescence ratio K’ was computed following the equations mentioned above [see 42 for derivation]. The transformed fluorescence ratio K’ was highly consistent among triplicates both for the cDNA and gDNA. The coefficient of variation (CV) of K’ was between 0.01% and 3.17% for cDNA and between 0.04% and 1.28% in gDNA (Table 1). The average CV (Intra-CV) for cDNA and gDNA was 0.70% and 0.45%, respectively. The standard curve was constructed based on K’ values and the predefined ratios of heterozygous plasmids (Fig 2). Values for single pure plasmids (either only *P*. *mexicana*-derived or only *P*. *latipinna*-derived) are included in Table 1. However, they did not fit well to the standard curve and were not considered in Fig 2, given the known heterozygous state of *P*. *formosa*. Both standard curves for cDNA and gDNA assessments exhibited a strong linear relationship among 11 standard points (R^2^> 0.99).

**Table 1:** The transformed fluorescence ratio K’ and associated coefficients of variation (CV, %).

	cDNA run		gDNA run	
Standard (Me: La)	K'	CV (%)	K'	CV (%)
**100**	0.705	0.42%	0.708	0.64%
**95**	0.684	0.02%	0.695	1.12%
**90**	0.657	0.33%	0.674	0.11%
**80**	0.612	0.33%	0.634	0.55%
**70**	0.584	0.07%	0.601	1.28%
**60**	0.556	0.01%	0.581	0.07%
**50**	0.522	0.38%	0.551	0.53%
**40**	0.495	0.36%	0.527	0.08%
**30**	0.459	3.17%	0.501	0.28%
**20**	0.440	0.26%	0.475	0.72%
**10**	0.414	0.47%	0.454	0.32%
**5**	0.403	0.29%	0.434	0.04%
**0**	0.420	2.98%	0.426	0.15%
**Intra CV (%)**		0.70%		0.45%

Standard: Premixed *P*. *mexicana*-derived and *P*. *latipinna*-derived plasmids according to the indicated, percentages x of Me (percentage of La 1-x).

K’: the average of transformed fluorescence ratio of all replicates.

Intra CV (%): the average coefficient of variation of 13 standards, indicating the accuracy of prediction.

Abbreviations in table, Me: *P*. *mexicana*-derived allele, La: *P*. *latipinna*-derived allele.

### Allele specific expression (ASE) at the androgen receptor alpha gene

**Fig 3:**
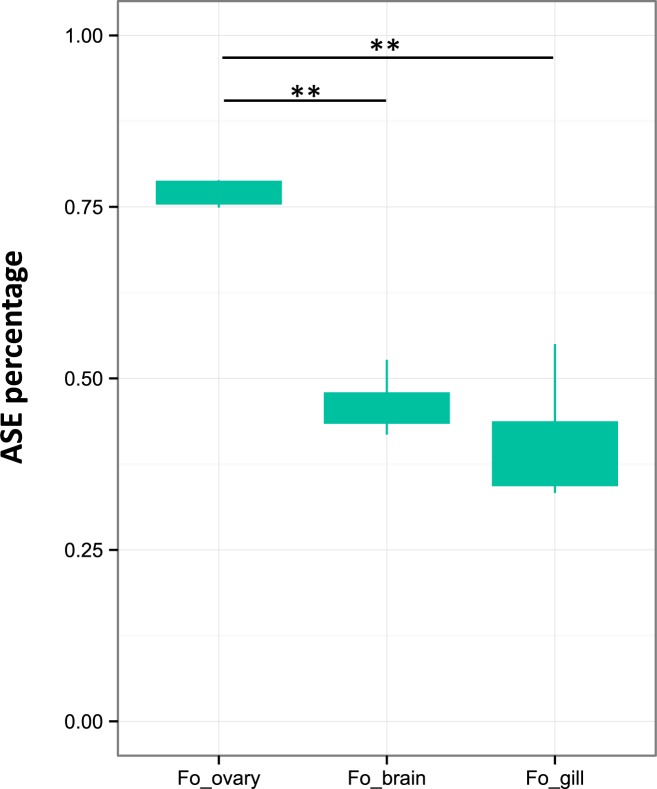
Allele specific expression (ASE) of *androgen receptor alpha* in *Poecilia formosa*. Percentages of the expression of the *P*. *mexicana*-derived allele in three tissues (ovary, brain, and gill) of *P*. *formosa* are presented. 0.5 indicates an equal (i.e., 1:1) expression of both alleles.Statistical significance was evaluated with an Analysis of Variance (ANOVA) and pairwise post-hoc-tests (** P< 0.001).

To investigate ASE of *arα* in *P*. *formosa*, one heterozygous marker (site 1974) was evaluated using Real-Time PCR. The mixes of plasmids (*P*. *mexicana*-derived allele and *P*. *latipinna*-derived allele) were used as standard curve and ASE calculation was conducted using the transformed fluorescence ratio K’. The ASE ratios (*P*. *mexicana*-derived allele to *P*. *latipinna*-derived allele) in ovary, brain, and gill in *P*. *formosa* are presented in Fig 3. The mean relative frequency of the *P*. *mexicana*-derived allele in *P*. *formosa* ovary was up to 0.770 (sd = 0.02), while these percentages in brain and gill were 0.462 (sd = 0.04) and 0.407 (sd = 0.09), respectively (Fig 3). Hence, for brain and gill, relative allele frequency did never significantly differ from 1:1, which we interprete as indication of equal expression. Compared to that, the significant deviation from 1:1 in ovary is indicative of allelic bias (higher expressed *P*. *mexicana*-derived allele than *P*. *latipinna*-derived allele). In our study, this allelic bias in ovary was reproducible among all four biological replicates.

The frequency of the two heterozygous alleles in the gDNA assessment ranged from 0.48 to 0.54 (*P*. *mexicana*-derived allele to *P*. *latipinna*-derived allele). They never significantly deviated from a 1:1 ratio.

## Discussion

In this study, we sequenced the upstream region of the transcription start site of *arα* in the hybrid all-female species *P*. *formosa* and its bisexual ancestral species, *P*. *mexicana* and *P*. *latipinna*. Consistent with the CDS results, the alignment of promoter regions of all species clearly demonstrated that *P*. *formosa* carries two different alleles of the promoter region, one of which derived from each of its two ancestors, *P*. *mexicana* and *P*. *latipinna*. The two bisexual ancestral species showed fixed single alleles in our laboratory strains. The two alleles were distinguished by one single nucleotide polymorphism and one indel. Interestingly, the indel site was located in a CpG island of *arα*.

There is evidence that polymorphisms at CpG sites may affect overall expression levels and allele specific expression. As an example, two polymorphic sites in a CpG island of the *CYP1A2* gene in human liver were found to strongly correlate both with mRNA expression levels and ASE phenotype [24]. To further evaluate the tissue-specific allelic bias in *P*. *formosa* ovary, therefore, the gene regulatory implications of the observed sequence variation in the putative promoter region warrants further investigations to arrive at a better understanding of *cis*-regulation of ASE. Our prediction of CpG islands and CpG sites may constitute a valuable resource for methylation studies. The association of SNPs among three species provided in this study with experimentally verified DNA methylation sites in promoter regions and proximal TSS regions would provide more information to understand the regulation of mRNA expression and may clarify, whether ASE is *cis*-regulated.

The transformed fluorescence ratio K’ method proved to accurately quantify the allele frequency in pooled DNA samples [42]. However, the accurate quantification of ASE heavily relies on the establishment of a correct standard curve. Here, it is essential to accurately mix the two alleles in the desired ratios. In our study, the most accurate concentration detection method, Qubit® dsDNA HS Assay, was used for concentration measurements of the plasmids carrying two different alleles (*P*. *mexicana*-derived allele or *P*. *latipinna*-derived allele). We applied the same method to gDNA of *P*. *formosa*, where–given the heterozygous status of this species [7,12–14]–the ratio is predicted to be 1:1. Indeed, in the gDNA assessment in this study, the heterozygous allele frequency ranged from 0.48 to 0.54, as expected. In fact, these results also suggest that there is no difference in gene copy number between the two ancestral species.

All unisexual vertebrates seem to be of hybrid origin [45] and have highly unusual reproduction, including sperm dependent parthenogenesis [1]. A fixed heterozygous state as the result of a hybrid origin was found in several vertebrates, including *P*. *formosa* [7,12,13,46–48]. Lampert and Schartl reported that the combination of two genomes with different mutations would result in the compensation of deleterious alleles, to the benefit of the hybrid [12]. Here, we utilized a single allele-specific SNP marker to investigate the differential expression of two different alleles of *arα*. ASE in *P*. *formosa* was estimated in several tissues, i.e., ovary, brain, and gill, using Real-Time PCR. Brain and gill did not show significant differential allelic expression. However, in ovarian tissue, the ASE of the two alleles showed a ratio of 7 to 3 (*P*. *mexicana*-derived to *P*. *latipinna*-derived), i.e., the allele derived from the maternal ancestor *P*. *mexicana* was expressed at level about twice as high as the other allele. A previous study on triploid Medaka (*Oryzias latipes*) reported that the maternally inherited allele was never silenced or suppressed, while one paternal allele was completely suppressed in the liver [23]. In cattle, tissue-specific complete suppression of either the maternally or paternally derived allele has been found [22]. In our present study, the maternal allele (*P*. *mexicana*-derived) exhibited higher expression than the paternal allele (*P*. *latipinna*-derived) only in ovarian tissue. Mechanisms and functions of tissue-specific ASE are still not well understood [49]. However, finding ASE specifically in ovarian tissue is an interesting starting point for future work. The ovary is not only the organ of gamete production, but also a major site of sex hormone synthesis and it also participates in hormonal regulation and endogenous interaction via the brain-ovary axis. Therefore, to better understand ASE in *arα* we suggest tissue-specific epigenetic studies and investigations of methylation sites in the putative promoter region of *arα* among tissues.

Previous studies indicate that one cannot generally assume a correlation between total mRNA expression and ASE, as, e.g., shown for the *CYP1A2* gene in human liver [24]. Also in our species, total mRNA expression and ASE of the *arα* gene are unrelated: While total expression was similar among ovary and brain, but less in gill [15], allelic bias was only detected in the ovary, but not in brain and gill tissue. In some hybrid systems, the interaction of the two alleles can either be additive or one allele can dominate the other [24,50–52]. ASE can increase the level of total gene expression, decrease it, or leave it unchanged. In a previous gene expression study in *P*. *formosa* [15], the total mRNA of *arα* in the ovary of *P*. *formosa* did not show a statistically significant difference relative to its ancestral species. However, those data revealed for ovary tissue some tendency towards higher expression in *P*. *latipinna* and lower expression in *P*. *mexicana*, with intermediate expression of *P*. *formosa*. Interestingly, in our study on the all-female *P*. *formosa*, the *P*. *mexicana*-derived allele (i.e., the allele from the maternal ancestor) is more expressed than the *P*. *latipinna*-derived allele (i.e., the allele from the paternal ancestor) specifically in the female gonad. As we identified potentially functionally relevant differences in the upstream putative promoter regions of the two alleles (see above), this allelic bias could constitute a *cis*-regulatory divergence [53]. Perhaps, a transcription factor specifically expressed in the ovary preferentially activates the *P*. *mexicana*-derived allele and hence causes the observed allelic bias.

Allelic bias in interspecies hybrids has been thoroughly investigated in the teleost fish genus *Xiphophorus* [54–56]. In these studies, allelic bias in the hybrid could—for some genes—be attributed to an allelic bias already present in the parental species, such that the hybrid expression patterns reflected parental ASE. Whether this is the case also regarding the allelic bias specifically occuring in the expression of the *arα* in *P*. *formosa* could, however, not be evaluated in the context of this study, as the specimens analyzed so far from both parental species were fixed for respective species-specific alleles with identical coding and promoter region.

As ASE does not have a significant impact on the total mRNA expression in *P*. *formosa*, both alleles are apparently still suited to serve the biological function of this gene. To our knowledge, this is the first study of ASE in a clonal, hybrid vertebrate. Our data provides an example of tissue-specific allelic bias in a species characterized by a combination of two genomes of fundamentally different origin. It would be interesting to discover how widespread allelic bias is in the transcriptomes of this and other clonal species.

## Supporting information

S1 Fig Polymorphic nucleotide positions (A) and the prediction of CpG islands and CpG sites (B) in the promoter region of androgen receptor alpha.
(PDF)
