# Supplementary material for: Allele-specific expression at the androgen receptor alpha gene in a hybrid unisexual fish, the Amazon molly (Poecilia formosa)
Source: PLoS One. 2017 Oct 12;12(10):e0186411. doi: 10.1371/journal.pone.0186411 (PMC5638567; doi:10.1371/journal.pone.0186411)
Supplement: S1 Fig — Polymorphic nucleotide positions (A) and the prediction of CpG islands and CpG sites (B) in the promoter region of androgen receptor alpha. (PDF) [file pone.0186411.s001.pdf]

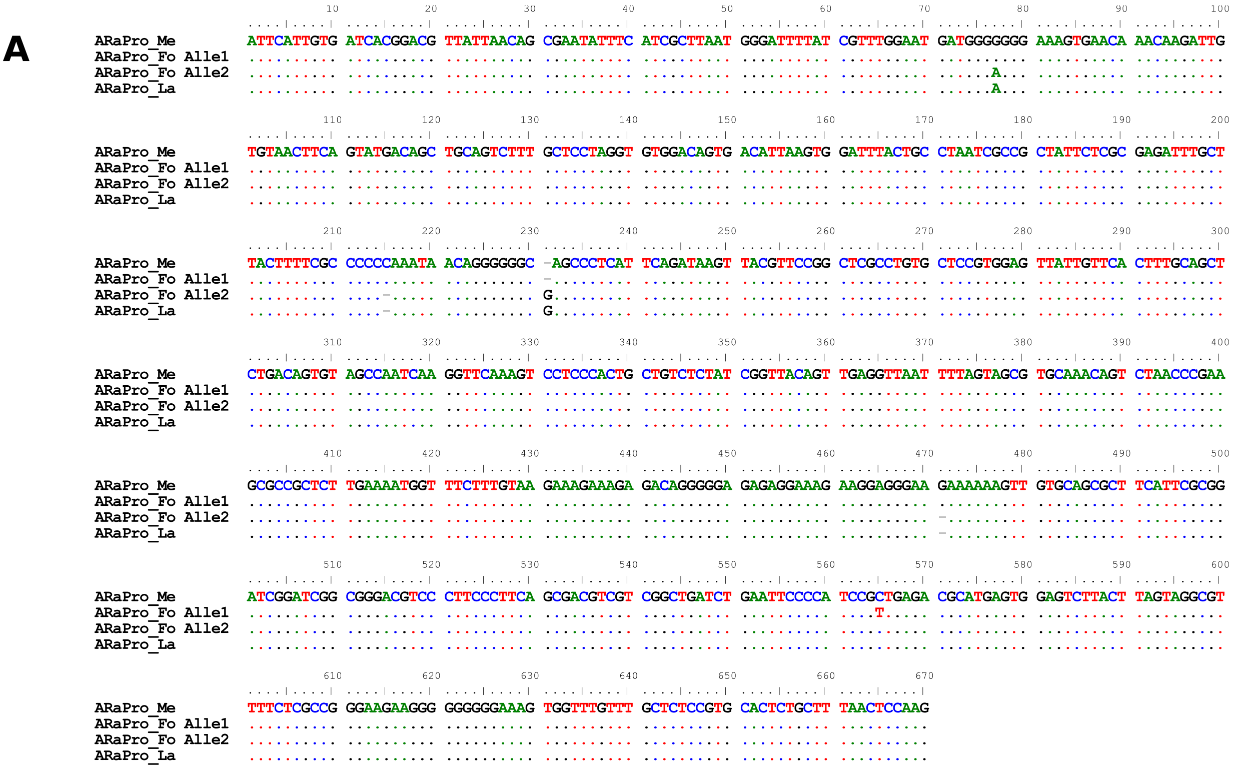

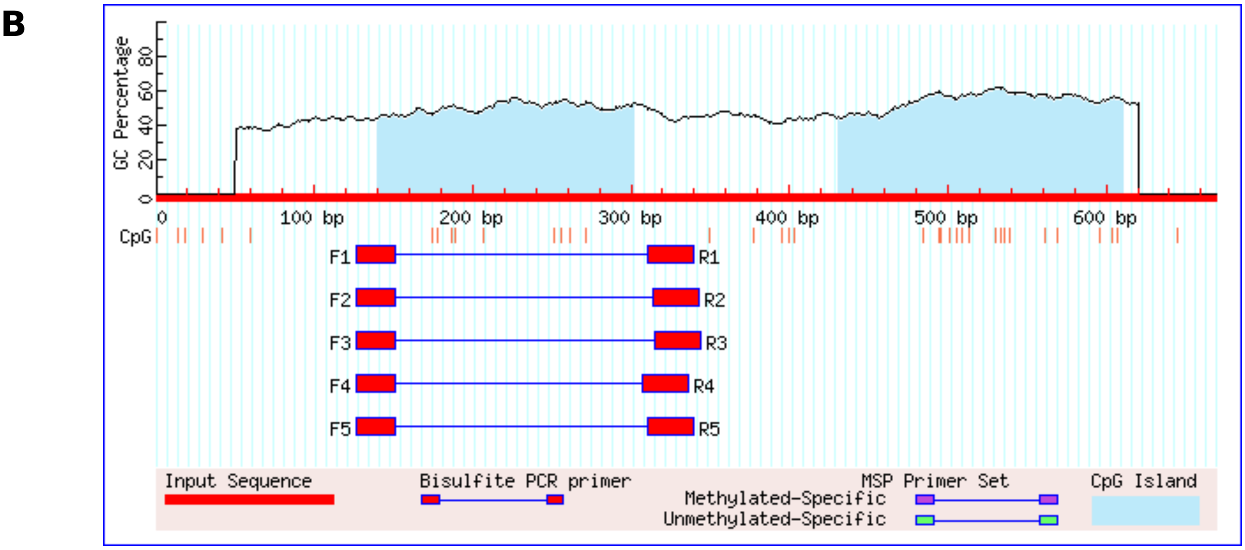

Supplementary Fig.1. Polymorhpic nucleotide positions (A) and the prediction of CpG islands and CpG sites (B) in the promoter region of androgen receptor alpha.
